# Keeping in Shape the Dogma of Mitochondrial DNA Maternal Inheritance

**DOI:** 10.1371/journal.pgen.1005179

**Published:** 2015-05-14

**Authors:** Valerio Carelli

**Affiliations:** 1 IRCCS Institute of Neurological Sciences of Bologna, Bellaria Hospital, Bologna, Italy; 2 Neurology Unit, Department of Biomedical and NeuroMotor Sciences (DIBINEM), University of Bologna, Bologna, Italy; Max Planck Institute for Biology of Ageing, GERMANY

## The Dogma: Uniparental (Maternal) Inheritance of mtDNA

It is textbook knowledge that the small multicopy mitochondrial genome (mtDNA) is maternally inherited in humans and mammals [1,2]. The uniparental mtDNA inheritance applies to most eukaryotic organisms, including animals exhibiting the doubly uniparental inheritance, such as the bivalve mollusks [3,4]. Occurrence of paternal mtDNA transmission has also been documented [5–7], and doubts on strict maternal inheritance in humans have been raised [8,9]. The best-documented case of paternal mtDNA inheritance was in a patient carrying a pathogenic mtDNA mutation [9], never replicated in following studies of patients with mitochondrial diseases due to various mtDNA defects [10–12].

The sperm mitochondria enter the oocyte during fertilization in mammals [13], but paternal mitochondria and mtDNA disappear at the initial cell divisions of the embryo in a stringently species-specific fashion [14]. In fact, the failure to efficiently eliminate paternal mtDNA from different species intercrosses [14,15] explains some of the cases of paternally inherited mtDNA [5]. Furthermore, recognition and targeted elimination of exogenous mtDNA entering the oocyte seems restricted to sperm mtDNA, not occurring with liver mtDNA, thus also displaying tissue specificity [16].

The way by which paternal mtDNA inheritance fails to occur in humans remains elusive, and it appears that several mechanisms have coevolved to avoid paternal mtDNA contribution to the embryo [17]. It has been observed that sperm mitochondria are ubiquitinated, suggestive of an “active elimination model” for paternal mtDNA [14], which may occur through different routes, such as proteosomal or lysosomal pathways [14,17]. Autophagy has been recently highlighted as the mechanism for paternal mtDNA elimination in *Caenorhabditis elegans* [18,19]. This was not observed in mice, for which elimination of mtDNA from prefertilization sperm and uneven persistence of paternal mtDNA in the embryo raised the possibility of a passive “dilution model” of disproportionate paternal versus maternal mtDNAs in mammals [20]. The consequent leakage of paternal mtDNA in the newborn may have remained “undetected” by the standard sequencing approaches.

## The “Dilution Model” Tested in Humans

Taking advantage of deep sequencing techniques, Pyle and colleagues tackled the issue of detectability of diluted postfertilization paternal mtDNA in humans [21]. They first estimated a ratio of 1:15,860 for the amount of mtDNA in healthy human sperm and prefertilization oocytes, predicting an interval for the proportion of the paternal haplotypes at fertilization of 10^–5^ to 1.8 x 10^–4^. Then, these authors went on using extremely high-depth mtDNA resequencing, up to about 1.2 million-fold coverage, to screen trios where the father and the child had two or more variant differences within a <200 bp stretch of mtDNA, looking for paternal haplotypes at very low heteroplasmy in buccal-derived DNA. A long-template strategy was used to generate the amplicons for resequencing, minimizing the artifactual identification of mitochondrial pseudogene variants in the “nuclear mitochondrial DNA” (NUMTs). Four different trios suitable to such analysis were identified out of a pre-existing cohort, and the analysis revealed the occurrence of extremely rare variant haplotypes, which were not compatible with a paternal origin and were thus considered as “background noise.” Most importantly, this “noise” was observed also in the maternal samples and was consistent within trios, raising the possibility of very low level contamination occurring when the original samples were acquired. Overall, this “noise” was incorporated into the statistical analysis and did not change the study conclusions that there is no evidence for paternal mtDNA contribution in the child.

## Is Buccal-Derived DNA Enough to Reject the “Dilution Model”?

This accurate study substantially rejects the hypothesis of a “dilution model” for paternal mtDNA transmission in humans [21]. The only debatable point remains the lack of a similar analysis in multiple tissues from the child. The confirmation of no paternal mtDNA haplotypes in multiple tissues, including postmitotic tissues, from the same individual would strengthen the current results, and there are a few reasons for this. According to Luo and colleagues [20], the skewed persistence of paternal mtDNA in only one of the 4-cell blastomers followed by subsequent uneven distribution to just a few cells at the morula stage of mouse embryos, would potentially lead to detectable paternal mtDNA only in some tissues of the newborn. Furthermore, age and tissue-dependent preferential shifts of one mtDNA haplotype over the other have been documented in heteroplasmic mice carrying a mixture of BALB and NZB mitochondrial genomes [22], potentially applying to the greatly disproportionate paternal versus maternal mtDNA ratio in the newborn tissues according to the “dilution model.”

## Is Maternal Inheritance Selected to Avoid Heteroplasmy?

The key question of why uniparental (maternal) mtDNA inheritance has been evolutionarily successful remains to be convincingly answered. The quick answer that sperm mtDNA is damaged by oxidative stress, being thus of bad quality and unfit to contribute the mtDNA pool of the embryo, is unsatisfactory. Maternal mtDNA inheritance avoids the occurrence of heteroplasmy between potentially distant mtDNA haplotypes, if coinherited by biparental mtDNA inheritance. The possible conflict between different coexisting “normal” mtDNA haplotypes, which may slightly differ in terms of oxidative phosphorylation (OXPHOS) efficiency, has been shown to be maladaptive in heteroplasmic mice, leading to significant physiological, cognitive, and behavioral impairments as compared to the homoplasmic mice for each mtDNA haplotype [23]. As a consequence, a non-random segregation of the mtDNA haplotypes occurs during tissue aging and germline transmission, leading to the proposal that this may explain the advantage of uniparental inheritance of mtDNA [23].

## Next: The “Active Elimination Model”

The study by Pyle and colleagues contributes to advance our understanding on how paternal mtDNA is not transmitted to newborns in humans [21]. The “active elimination model” takes over the “dilution model,” but besides ubiquitination of sperm mitochondria, we still do not know how their elimination is executed. Is ubiquitination targeting specific proteins? Prohibitin has been reported to be ubiquitinated in the sperm [24] and has also been proposed as the regulator of TFAM and mtDNA copy number [25]. In turn, reduction of TFAM and mtDNA copy number occurs during mammalian spermatogenesis [26]. Thus, a first step of a possibly multistep mechanism reduces sperm mtDNA to a minimal amount. Once the oocyte is fertilized, proteasomal and lysosomal pathways have been invoked for paternal mitochondria and mtDNA elimination. However, emerging aspects of mitochondrial quality control and dynamics [27] are poorly known in the contest of fertilized oocytes, which may turn out relevant to paternal mtDNA elimination. Finally, specific recognition and elimination of paternal mtDNA may occur at the molecular level. Endonuclease G has been implicated in reduction of sperm mtDNA copy number in *Drosophila* [28], but whether paternal mtDNA is directly targeted postfertilization remains unexplored.

## Conclusions

**Fig 1:**
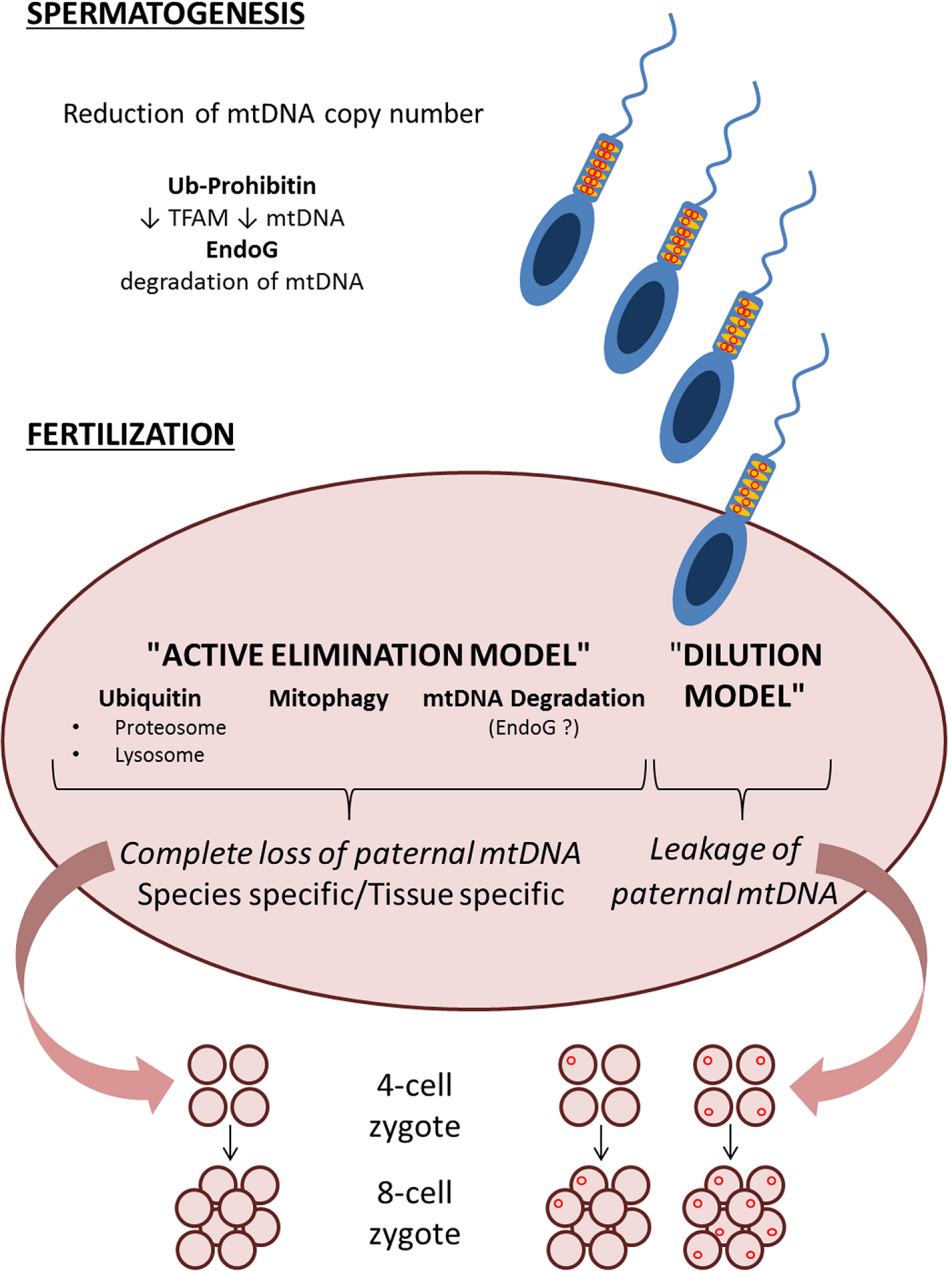
Schematic representation of the two models, “active elimination” and “dilution” of paternal mtDNA haplotypes, with multiple possible steps that ensure avoidance of paternal mtDNA inheritance. A first step for which there is evidence of reduction of mtDNA copy number is at the level of spermatogenesis and prefertilization sperm [20,24–26]. Postfertilization, according to the “dilution” model, the low levels of paternal mtDNA haplotypes may be evenly distributed among tissues, but the study by Pyle and colleagues finds no evidence of such a “dilution” [21]. Alternatively, if mtDNA haplotypes are unevenly distributed among the tissues of the newborn [20], or shift in an age and tissue-dependent fashion [22], there remains a possibility that paternal mtDNA is detectable only in certain tissues. The “active elimination” model, currently more supported by experimental evidence, may execute the paternal mtDNA elimination through multiple possible mechanisms, which are summarized in Fig 1. These include ubiquitination and active elimination of paternal mitochondria and mtDNA by proteasomal and lysosomal pathways [14], selective mitophagy of paternal mitochondria [18,19], or direct degradation of paternal mtDNA [28].

The emerging picture is that of a multistep mechanism, with many different checkpoints composing a puzzle (Fig 1) needing more work to be completed to fully unwrap the dogma of mtDNA maternal inheritance.
